# Linking spatial gene expression patterns to sex-specific brain structural changes on a mouse model of 16p11.2 hemideletion

**DOI:** 10.1038/s41398-018-0157-z

**Published:** 2018-05-29

**Authors:** Vinod Jangir Kumar, Nicola M. Grissom, Sarah E. McKee, Hannah Schoch, Nicole Bowman, Robbert Havekes, Manoj Kumar, Stephen Pickup, Harish Poptani, Teresa M. Reyes, Mike Hawrylycz, Ted Abel, Thomas Nickl-Jockschat

**Affiliations:** 1 Department of Psychiatry, Psychotherapy and Psychosomatics, RWTH Aachen University, Aachen, Germany; 2 Juelich-Aachen Research Alliance Brain, Juelich/Aachen, Germany; 3 Max Planck Institute for Biological Cybernetics, Tubingen, Germany; 4 Department of Pharmacology, University of Pennsylvania, Philadelphia, PA USA; 5 Institute for Translational Medicine and Therapeutics, University of Pennsylvania, Philadelphia, PA USA; 6 Department of Psychology, University of Minnesota, Minneapolis, MN USA; 7 Department of Biology, University of Pennsylvania, Philadelphia, PA USA; 8 Department of Neuroscience, University of Pennsylvania, Philadelphia, PA USA; 9 Groningen Institute for Evolutionary Life Sciences, University of Groningen, Groningen, Netherlands; 10 Department of Radiology, University of Pennsylvania, Philadelphia, PA USA; 11 Centre for Preclinical Imaging, University of Liverpool, Liverpool, UK; 12 Department of Psychiatry and Behavioral Neurosciences, University of Cincinnati, Cincinnati, OH USA; 13 Allen Institute for Brain Science, Seattle, WA USA; 14 Iowa Neuroscience Institute, Carver College of Medicine, University of Iowa, Iowa, IA USA; 15 Department of Psychiatry, Carver College of Medicine, University of Iowa, Iowa, IA USA

## Abstract

Neurodevelopmental disorders, such as ASD and ADHD, affect males about three to four times more often than females. 16p11.2 hemideletion is a copy number variation that is highly associated with neurodevelopmental disorders. Previous work from our lab has shown that a mouse model of 16p11.2 hemideletion (del/+) exhibits male-specific behavioral phenotypes. We, therefore, aimed to investigate with magnetic resonance imaging (MRI), whether del/+ animals also exhibited a sex-specific neuroanatomical endophenotype. Using the Allen Mouse Brain Atlas, we analyzed the expression patterns of the 27 genes within the 16p11.2 region to identify which gene expression patterns spatially overlapped with brain structural changes. MRI was performed ex vivo and the resulting images were analyzed using Voxel-based morphometry for T1-weighted sequences and tract-based spatial statistics for diffusion-weighted images. In a subsequent step, all available in situ hybridization (ISH) maps of the genes involved in the 16p11.2 hemideletion were aligned to Waxholm space and clusters obtained by sex-specific group comparisons were analyzed to determine which gene(s) showed the highest expression in these regions. We found pronounced sex-specific changes in male animals with increased fractional anisotropy in medial fiber tracts, especially in those proximate to the striatum. Moreover, we were able to identify gene expression patterns spatially overlapping with male-specific structural changes that were associated with neurite outgrowth and the MAPK pathway. Of note, previous molecular studies have found convergent changes that point to a sex-specific dysregulation of MAPK signaling. This convergent evidence supports the idea that ISH maps can be used to meaningfully analyze imaging data sets.

## Introduction

Copy number variations (CNVs) play a pivotal role in the genetic architecture of neurodevelopmental disorders^1^. An excess of comparatively large and rare genomic hemideletions or hemiduplications has been reported consistently in individuals affected with, e.g., autism spectrum disorder (ASD)^2–5^ or attention deficit/hyperactivity disorder (ADHD)^6–9^. Although these mutations often go along with significantly increased odds ratios for the manifestation of psychiatric and neurodevelopmental disorders^5,9,10^, the relationship between genetic variation and disease is often complex. CNVs are usually not disease-specific, but rather significantly increase the manifestation risk for several disorders^1^. This renders them ideal candidates to model basic pathophysiological processes across diseases.

A pronounced male excess of around 3–4:1 in ASD or ADHD is one of the most consistent epidemiological findings^11–18^. This male preponderance with almost the same male–female ratio across several neurodevelopmental disorders has led to speculations that a shared mechanism might underlie gender-specific increased vulnerability^16–19^. The 16p11.2 hemideletion contains 27 genes and is significantly associated not only with increased risk for ASD, ADHD, and intellectual disability (ID), per se^19^, but also with increased male vulnerability at least to ASD and ID^20^. Given that the genetic architecture of this chromosomal region is highly conserved, the human variant can be modeled very closely in mice via hemideletion of chromosome 7qF3^21^. In our recent work, we have found male-specific deficits in reward learning^22^ and sleep patterns^23^ in a mouse model for 16p11.2 hemideletion. These male-specific behavioral phenotypes make this mouse model a unique opportunity to study the underlying mechanisms of sex-specific vulnerability to neurodevelopmental disorders.

Brain structural changes are commonly regarded as relevant factors in the pathogenesis of neurodevelopmental disorders^24,25^ that relate to the symptoms level^26,27^. These structural changes are observed not only in patients with ASD^28^ and ADHD^29^ but also in samples of human 16p11.2 hemideletion carriers across diagnoses^30,31^. The latter finding suggests a causal role for 16p11.2 hemideletion in neuroanatomical anomalies. Structural imaging in an animal model of a genetic lesion can serve as a powerful translational tool to investigate these neuroanatomical changes under standardized conditions and help to detect sex-specific mechanisms that are conserved across species^32–35^.

In studies of single-gene mutations, it is easier to make a direct inference that observed phenotypes are due to the deletion of that particular gene^36^. The 16p11.2 hemideletion, in contrast, involves 27 genes. Given the diverse biological functions of their gene products^34^, one hypothesis is that individual hemideleted genes contribute to different degrees to the brain structural changes. Thus, even if robust neuroanatomical differences were to be detected between a 16p11.2 hemideletion mouse model and wild types, it still remains to be determined which of these 27 are associated with these neuroanatomical changes. Innovative analytical approaches are needed to address this problem. Modern gene expression atlases are powerful tools that have the potential to yield answers for this dilemma. The Allen Mouse Brain Atlas (http://mouse.brain-map.org) is an ideal open-access resource that enables the user to quantify gene expression at a resolution of 200 µm^3^ across the entire P56 mouse brain^37^. These genes maps, however, are not in a standard imaging space^38^. If they could be aligned to a standard imaging template brain, a voxel-wise assessment of gene expression patterns could be related to brain structural changes detected by magnetic resonance imaging (MRI) nearly 1:1. Based on the idea that genes whose expression patterns spatially overlap with brain structural changes are plausible candidates to mediate these changes, this approach could be used to generate observer-independent hypotheses on putative relationships between gene expression and structural endophenotypes. In other words: genes, whose spatial expression patterns largely overlap with brain structural changes are more likely involved in the pathogenesis of these changes than those genes that do not show this pattern. It should be noted that such an approach certainly does not allow for causal inferences between gene expression patterns and brain structural changes, especially because the gene expression maps provided by the Allen Mouse Brain Atlas were acquired at P56. Although changes in gene expression might well influence brain structure also long after the embryonic period, our correlative analysis does not allow a straightforward interpretation, whether gene expression patterns influenced the formation of brain structural changes or whether more complex mechanisms underlie this association.

For our MRI study on sex-specific changes in a mouse model of 16p11.2 hemideletion reported here, we used the Allen Mouse Brain Atlas to evaluate which genes within the deletion region were overexpressed in the structurally altered regions compared to the rest of the brain. Genes overexpressed in regions of the male del/+ endophenotype were associated with neurite outgrowth and the MAPK pathway, two plausible mechanisms for the pathogenesis of neurodevelopmental disorders.

## Materials and methods

### Animals

All animals were cared for in accordance with the guidelines of the National Institutes of Health and were approved by the University of Pennsylvania and Washington State University Institutional Animal Care and Use Committees. Colony founders from the 16p11.2 hemideletion (del/+) line generated by the laboratory of Dr. Alea Mills on a C57BL/6J and 129S1/SvImJ F1 background were obtained from Jackson Laboratories (male founders: B6129S-Del(7Slx1b-Sept1)4Aam/J; Jackson Laboratories Stock # 013128; female founders: Females; B6129SF1/J; Jackson Laboratories Stock #101043). Before animals underwent imaging, they were genotyped to make sure they were assigned to the right experimental group.

### Mouse brain preparation

We performed all MRI scans ex vivo. Wild-type and hemideleted mice were sacrificed at the age of 70 days. In total, 9 (del/+) mutants (4 males and 5 females) and 12 wild types (6 males and 6 females) were included in the study. No animals were excluded. Sample sizes were chosen according to previous behavioral findings of our lab^22^. Animals were chosen at random for the experimental groups. Their brains were perfused and fixated. The brains were kept in 4% paraformaldehyde solution for 4 days to achieve optimal T1/T2 relaxation time^39^.

### Image acquisition

Imaging was performed on a 9.4 T animal scanner with a custom-built solenoid coil (20 mm inner diameter) at the University of Pennsylvania, Philadelphia, PA, USA. The investigator was blind to the experimental group during the scan. A structural T1-weighted MRI sequence was acquired at a resolution of 39 × 39 × 39 micron (acquisition time: 1 h 50 min). Diffusion tensor images were acquired with a diffusion-weighted (DW) sequence with TR/TE = 800/29.50 ms and a *b*-value = 902 mm^2^/s (acquisition time = 13 h). The image resolution was 125 µm isotropic. The image size was 136 × 68 × 80. Six diffusion directions were acquired.

### Image preprocessing

The scanner-generated fid signals were converted into NIFTI image format using MRIstudio (https://www.mristudio.org/). The quality of the format conversion was visually inspected. Since the FSL software^40^ was programmed for the analysis of human magnetic resonance (MR) data, it fails to process ultra-high-resolution structural MRI (39 microns isotropic) images that we acquired in this study. Therefore, we modified the image headers to 1 × 1 × 1 mm, but the raw data matrix remained unchanged. This allowed seemingly smooth further data processing.

### Voxel-based morphometry

Gray matter segmentation and volumetric analysis of the structural images were carried out using registration and permutation test scripts from FSL and tract-based spatial statistics (TBSS)^41–43^.

MR gray matter segmentation for human data sets uses probability maps of gray and white matter tissue types as priors. However, in our mouse data, so far, no such high-resolution tissue priors exist. Therefore, we relied upon an intensity-based segmentation using FSL tools^40^, rather than the conventional SPM-based standard protocol^44^. Quality of the white matter removal was visually inspected.

Accurate gray matter volume detection is very sensitive to the quality of the registration to a given template brain. Therefore, an optimized alignment is essential for accurate results. To minimize the average warping for all data sets (cf. ref. ^41^), we first aligned all brains to each other and identified the “most typical” brain of the sample. This “most representative brain” was identified by aligning all T1-weighted data sets to each other, estimating the average amount of warping necessary and then picking the one that had the smallest amount of average warping when used as a target (https://www.fmrib.ox.ac.uk/datasets/techrep/tr07ja2/tr07ja2.pdf). In the next step, we applied the nonlinear transformations found in the previous step to all structural images to bring them into reference space.

Statistical comparisons between wild-type vs del/+ animals were conducted for both sexes separately. Contrasts were conducted in both directions using two-group unpaired *t*-tests with 500 permutations. Test statistics were computed using threshold-free cluster enhancement (TFCE) as implemented in FSL-randomize^45^. The following four contrasts were calculated: FA increases in male del/+; FA decreases in male del/+; FA increases in female del/+; and FA decreases in female del/+.

### TBSS analysis

For our analysis of the DW data sets, we opted for the TBSS approach^41,42^ as the best established analysis method for this imaging modality, using FSL tools^42,46^ (http://www.fmrib.ox.ac.uk/fsl/).

To run TBSS on mouse data sets, the image headers were modified to 1 × 1 × 1 mm isotropic resolution, but the raw data matrix was left unchanged. This modification helps TBSS to deal with the same data efficiently without any alteration in the actual resolution of the data sets. We used fractional anisotropy (FA) maps as a standard and well-interpretable diffusion tensor imaging (DTI) parameter for all further analyses.

TBSS was designed for in vivo human DTI images and proposes an FA threshold of ≥0.2 to exclude voxels situated outside the White Matter^41,42^. However, both the regional and the quantitative distribution patterns of FA values of these data sets can be reasonably expected to differ from those in postmortem mouse DW images. To avoid distortions of our findings due to these factors, we applied different FA thresholds to our maps and visually inspected the resulting fiber tract skeletons. To our surprise, the standard FA threshold ≥0.2 yielded the optimal results. We, therefore, applied this as threshold for all subsequent steps.

After removing the outliers and the zero end slices from the FA maps, individual fiber tracts were extracted and aligned to the target map, i.e., the individual fiber tract skeleton of the group that shows the smallest amount of average warping (see above). After a successful alignment was ascertained by inspecting each step visually, voxel-wise statistical comparisons between wild-type vs del/+ animals were conducted for both sexes separately using two-group unpaired *t*-test (500 permutations). We used TFCE at a threshold of *p* < 0.05 to check for the following four contrasts, as we did in our voxel-based morphometry (VBM) analysis: FA increases in male del/+; FA decreases in male del/+; FA increases in female del/+; and FA decreases in female del/+.

### Transformation of gene expression maps provided by the Allen Mouse Brain Atlas to Waxholm space

We first downloaded all available in situ hybridization (ISH) gene expression maps of the 27 genes involved in the deletion (http://mouse.brain-map.org/) (Table 1). Unfortunately, we were not able to obtain a map for PRRT2, while all other 26 gene expression maps were available.

**Table 1:** Overview over the 27 genes involved in the deletion

Gene symbol	Gene name	Aliases	Plane	Experiment no.
*Spn*	Sialophorin	A630014B01Rik, Ly-48, Galgp, Cd43, Ly48	Sagittal	72002080
*Qprt*	Quinolinate phosphoribosyltransferase	AI647766, QPRTase, 2410027J01Rik	Sagittal	71617020
*Kif22*	Kinesin family member 22	AU021460, Kid, C81217, Kif22a	Sagittal	72726
*Maz*	MYC-associated zinc finger protein (purine-binding transcription factor)	Pur-1, PUR1, SAF-1, SAF-2	Sagittal	71488768
*Prrt2*	Not available in Allen			
*Pagr1a*	RIKEN cDNA 2900092E17 gene	*2900092E17Rik*	Sagital	68076546
*Mvp*	Major vault protein	LRP, VAULT1, 2310009M24Rik	Sagittal	275693
*Cdipt*	CDP-diacylglycerol–inositol 3-phosphatidyltransferase (phosphatidylinositol synthase)	D7Bwg0575e, 9530042F15Rik, Pis, Pis1	Sagittal	76098296
*Sez6l2*	Seizure-related 6 homolog like 2	AI835913, MGC19060, AW121566, Psk1, BSRP-A, MGC90604	Sagittal	1408
*Asphd1*	Aspartate beta-hydroxylase domain containing 1	MGC130534, Gm168, A830007L07Rik	Sagittal	69449026
*Kctd13*	Potassium channel tetramerization domain containing 13	Poldip1, PDIP1alpha, 1500003N18Rik, AV259508	Sagittal	71488716
*Tmem219*	Transmembrane protein 219	2700081K05Rik, 6330540D07Rik, mCG18160.2, CXorf44-like, LOC382245	Sagittal	70596194
*Taok2*	TAO kinase 2	MAP3K17, TAO1, KIAA0881, 1110033K02Rik, TAO2, PSK1, mKIAA0881, PSK, B230344N16	Sagittal	72081744
*Hirip3*	HIRA interacting protein 3	B130036O03, C86302	Sagittal	69837911
*Ino80e*	INO80 complex subunit E	Ccdc85, MGC31515, AI225782, AI854876, Ccdc95	Sagittal	67815968
*Doc2a*	Double C2, alpha		Sagittal	68861994
*Fam57b*	Family with sequence similarity 57, member B	AI413816, MGC103222, AW060769, A330104J06Rik, 1500016O10Rik	Sagittal	75831762
*AldoA*	Aldolase A, fructose-bisphosphate	Aldo-1, Aldo1, MGC107164	Sagital	77620804
*Ppp4c*	Protein phosphatase 4, catalytic subunit	AU016079, Ppx, 1110002D08Rik	Sagittal	632486
*Tbx6*	T-box 6	rv	Sagittal	71280631
*Ypel3*	Yippee-like 3 (Drosophila)	0610043B10Rik, 1190001G19Rik, Suap	Sagittal	75214942
*Mapk3*	Mitogen-activated protein kinase 3	Prkm3, Esrk1, p44, Erk-1, Erk1, p44erk1, p44mapk, Mnk1, Mtap2k, Ert2	Sagittal	71608206
*Coro1a*	Coronin, actin-binding protein 1A	Clabp, p57, TACO, Lmb3	Sagittal	67978734
*4930451I11Rik*	RIKEN cDNA 4930451I11 gene		Sagittal	75851059
*Zg16*	Zymogen granule protein 16		Sagittal	68632924
*AI467606*	Expressed sequence AI467606		Sagittal	71656675
*Gdpd3*	Glycerophosphodiester phosphodiesterase domain containing 3	1110015E22Rik	Sagittal	75694405

The first and the second columns of the table provide the gene symbol and the gene name as given by the Allen Mouse Brain Atlas, while the third column lists the most used alias names of these genes. The second column from the right gives an overview over the orientation of the ISH maps. We used sagittally oriented maps for all subsequent analyses. The rightmost column holds the identification number of the ISH experiment in the database of the Allen Institute. A gene expression map for PRRT2 was not available in the entire Allen Mouse Brain Atlas database

The Allen Mouse Brain Atlas provides two different types of information per slide: a Nissl staining and a gene expression map^37^. The most commonly used tool for visualization, the Allen Brain Explorer, displays the gene expression map of interest superimposed over the Nissl stainings (http://help.brain-map.org/download/attachments/2818169/AllenReferenceAtlas_v1_2008_102011.pdf?version=1&modificationDate=1319477213862). Aligning these gene expression maps and the individual MRI data sets to a common anatomical space, however, is a major hurdle for this type of analysis. Conventional alignment algorithms^43^ cannot be used on gene expression maps, since the contrast properties of the latter diverge distinctly from MRI data sets.

To address this crucial problem, we treated both data sets—i.e., the Nissl-stained images and the gene expression maps—separately. In a first step, we conducted a three-dimensional reconstruction and transformed the Nissl-stained images file (in the following in brief “Nissl NIFTI”) and each gene expression map into a NIFTI file.

In the next step, we obtained data sets from the Waxholm Space Atlas^38^, which consisted of T1w-, T2*-, and T2-weighted images (http://software.incf.org/software/waxholm-space/download). We then created registration matrices as follows:

- Allen low-resolution Nissl space (200 microns isotropic) to high-resolution Nissl space (25 microns): Allen energy (=gene expression) maps were acquired in a 200-micron isotropic resolution, and, therefore, they required up-sampling for a transformation into Waxholm Space. First, we ensured a similar orientation of both images. Second, we realized a 200-micron isotropic to 25-micron isotropic transformation using 12-parameter affine registration, search space of +90° in *x*,*y*,*z* directions and trilinear interpolation. This step is necessary to ensure a proper alignment of the gene expression maps (see below).
- The 25-micron isotropic Nissl stack was linearly aligned (12-parameter affine registration) to 25-micron isotropic gray-scaled Nissl space. This step was necessary because gray-scaled images provided a better contrast for the following registration to 21.5 isotropic T2-weighted Waxholm space.
- This gray-scaled Nissl image was linearly registered to a T2-weighted template in Waxholm space with 12-parameter affine registration.

In the above three steps, we saved the deformation fields from each step. The quality of each of the registrations was inspected visually after the alignment. Although the contrast properties of the Nissl NIFTI files certainly differed from an MRI image, the alignment to Waxholm space was highly precise. The use of T2-weighted Waxholm templates empirically resulted in better registration than the contrast properties of T1 and T2* images.

**Fig. 1:**
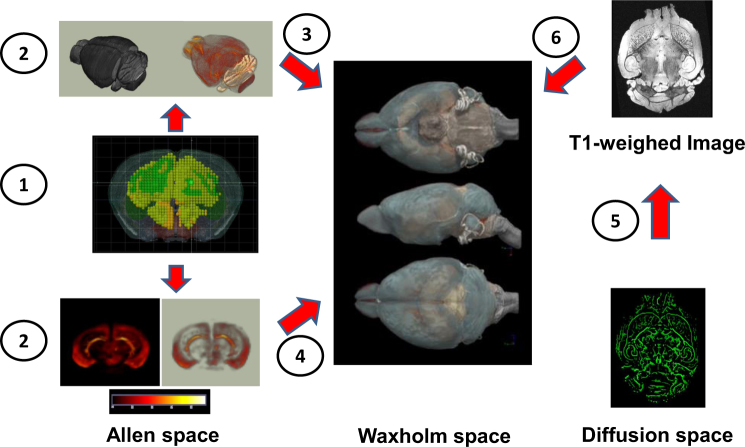
Schematic overview over the alignment process of ISH maps from the Allen Mouse Atlas to Waxholm space. The Allen Mouse Brain Explorer usually displays the gene expression map of interest superimposed over the Nissl stainings (1). Conventional alignment algorithms do not properly transform these images into a standard anatomical space. Therefore, both data sets—i.e., the Nissl-stained images and the gene expression maps—were treated separately and 3D reconstructed in a first step (2). We used linear registration algorithm to align the NIFTI of Nissl-stained slides to Waxholm standard space (3). We then used the resulting deformation field on the NIFTI files of each gene expression map and, thus, aligned them to Waxholm space (4). We next linearly registered the mean FA skeleton derived from our TBSS analysis to the corresponding skull-stripped T1-weighted brain scan of the same animal (5). The T1-weighted images were then registered to Waxholm space (6). This step enabled us to transfer the clusters obtained by our TBSS analysis from diffusion space to Waxholm space and directly compare them to the gene expression maps

**Fig. 2:**
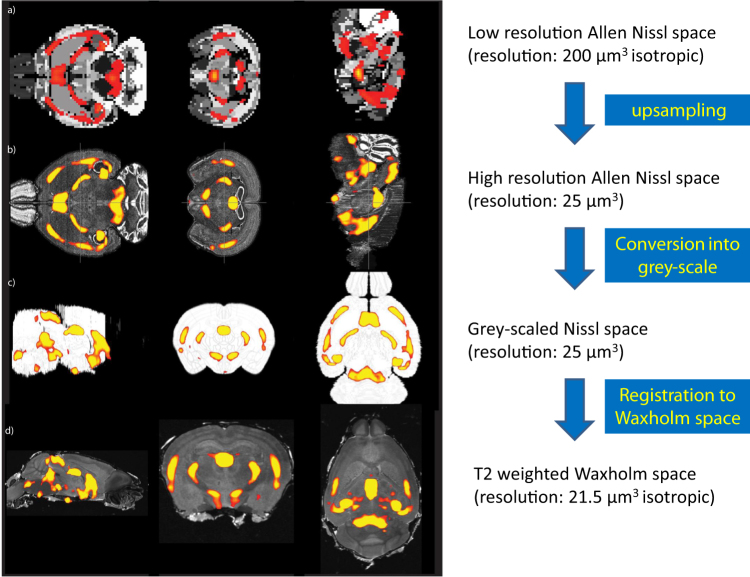
Overview over the different steps of creating registration matrices to adjust the images for the different resolution scales. This is a pivotal step to optimize conditions for a proper alignment process. Low-resolution Allen Nissl space (200 microns isotropic) (**a**) are up-sampled to high-resolution Nissl space (25 microns) (**b**). Nissl-stained images are then converted into gray-scaled Nissl space (**c**). This step was necessary because gray-scaled images provided a better contrast for the following registration to 21.5 isotropic T2-fweighted Waxholm space (**d**)

We then used the resulting deformation fields of the NIFTI file alignments of each gene expression (=energy) map and, thus, aligned them to Waxholm space. To check for potential distortions of gene expression values due to the alignment process, histograms of the gene expression maps were inspected before and after the transformation (Figs. 1 and 2).

As described above, in our TBSS analysis all FA skeletons were aligned to the “most typical one” of the entire group. We took this template FA map, and linearly registered it to the corresponding skull-stripped T1-weighted brain scan of the same animal. The T1-weighted data set was then registered to Waxholm space. This step enabled us to transfer the clusters obtained by our TBSS analysis from diffusion space to Waxholm space and directly compare them to the gene expression maps (Fig. 1).

### Code availability

Scripts for these deformations are publicly available (https://medicine.uiowa.edu/iowaneuroscience/research/computational-psychiatry/gene-expression-tool).

### Statistical analysis of genes overexpressed in the structurally altered regions compared to the rest of the fiber tract skeleton

We extracted expression values of all 26 gene maps overlapping with the clusters that stemmed from the four contrasts of our TBSS analysis (increased FA in male del/+, decreased FA in male del/+, increased FA in female del/+, and decreased FA in female del/+) and also from the entire FA skeleton. Gene expression values were pooled over all clusters from one distinct contrast and its distribution compared to gene expression values over the entire FA skeleton by using a Ranksum test. The Ranksum test is comparable to *t*-test, but also works efficiently on non-normal distributed data (http://de.mathworks.com/help/stats/ranksum.html). We used a threshold of *p* < 0.05 to identify genes that were significantly overexpressed in one of our contrasts compared to the entire FA skeleton.

## Results

### Sex-specific changes of white matter integrity in del/+ animals

Findings of increased FA in medial white matter regions have been described in human 16p11.2 hemideletion carriers^30^. Although DW imaging has been applied previously to a mouse model of the deletion^35^, detailed studies on white matter changes, however, are lacking up to now in mouse models of this deletion. We, therefore, aimed to investigate whether del/+ animals displayed similar changes as humans. Given a sex-specific behavioral phenotype with regard to deficits in reward learning, we chose to conduct separate analyses for both sexes comparing FA in both del/+ and wild-type animals. We used TBSS^41,42^ in an optimized version for mouse brains as gold standard for the analysis of DW imaging data sets.

**Fig. 3:**
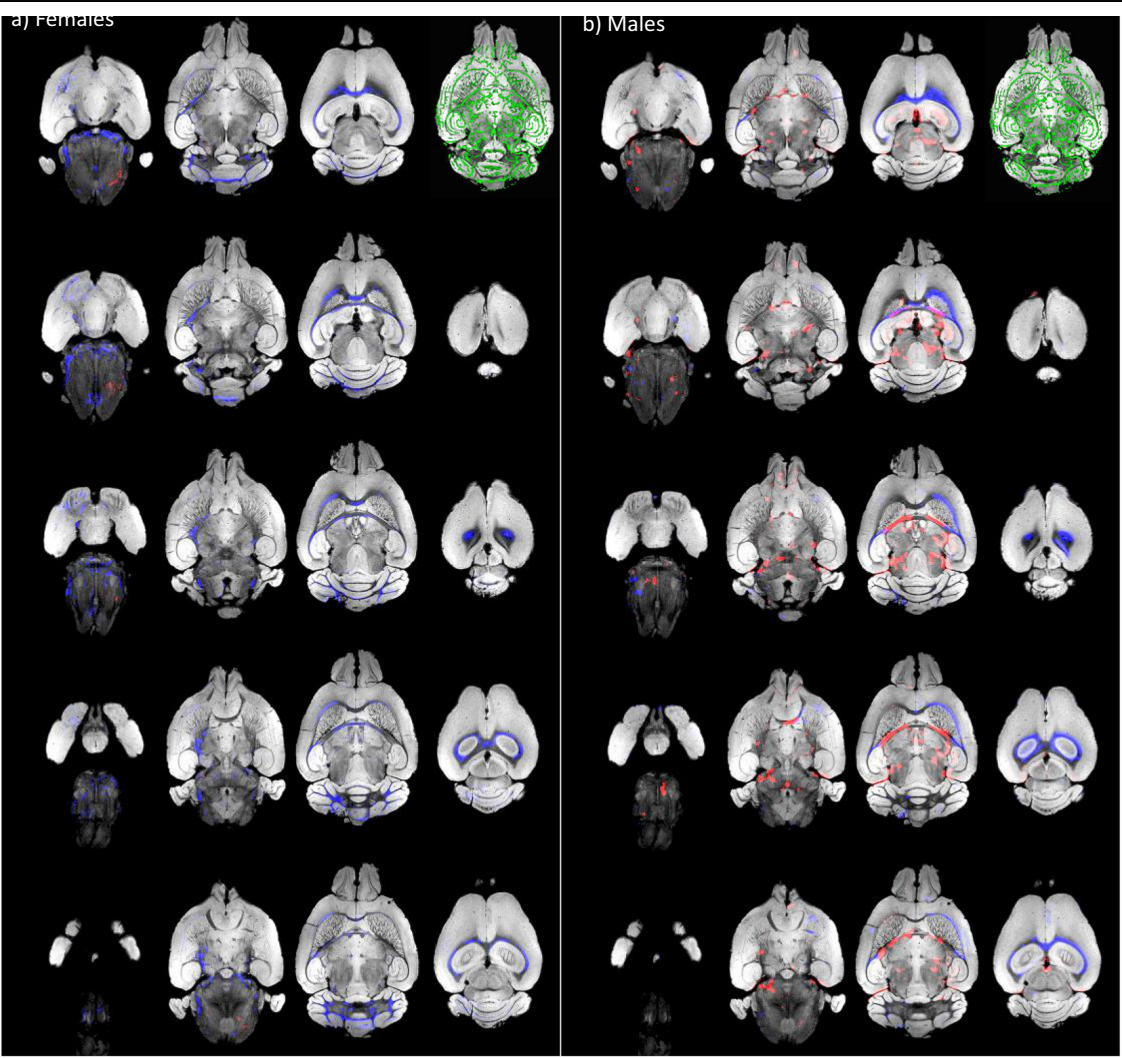
Fiber tract changes in female (left) and male (right) del/+ animals compared to wild types of the same sex. Increases of fractional anisotropy (FA) in the del/+ animals are displayed in red, FA decreases in blue. The mean fiber tract skeleton is displayed in green. We found widespread decreased FA in both male and female del/+. In female del/+, FA decreases were detectable in fiber tracts throughout telencephalic and cerebellar regions, with increased FA only in small cerebellar regions. In male del/+ animals, we found pronounced FA increases in medial and peristriatal fiber tracts

Our two-group unpaired *t*-test with 500 permutations using TFCE at a threshold of *p* < 0.05 retrieved widespread decreased FA in both male and female del/+ that involved most notably transcallosal fibers. In female del/+, decreased FA was prevalent in fiber tracts throughout telencephalic and cerebellar regions. Increased FA values were detectable only in small clusters in the cerebellum. In contrast to that we found pronounced FA increases in medial and peristriatal fiber tracts in male del/+ animals that constituted a distinct male endophenotype (Fig. 3).

As cortical anomalies were also observed in human 16p11.2 hemideletion carriers^30^, we conducted a VBM analysis^42,44^ to explore potential gray matter changes in del/+ animals, again comparing del/+ and wild-type animals of both sexes separately. However, at a significance threshold of *p* < 0.05, a two-group unpaired *t*-tests with 500 permutations did not yield any significant results for any of the computed contrasts.

### Analysis with gene expression maps allows the identification of genes specifically overexpressed in regions that constitute a male-specific endophenotype

In a next step, we aimed to explore which of the hemideleted genes were overexpressed in the structurally affected brain regions of del/+ animals. This approach is based on the hypothesis that genes showing expression patterns that spatially overlap with brain structure anomalies are the most plausible candidates to be causally related to these neuroanatomical changes. Given the sex-specific behavioral phenotypes^22,23^ and the male-specific structural endophenotype, we especially focused on genes overexpressed in the regions altered specifically in del/+ males.

We used gene expression maps downloaded from the Allen Mouse Brain atlas. These maps are based on ISH data and provide spatial information about the expression levels of a gene at a resolution of 200 µm^3^. Gene expression in the mouse brain varies in different major cell types and, thus, differs significantly between gray and white matter^37,47^. Given this, we chose to compare mean gene expression within the clusters to mean expression values averaged over the entire fiber tract skeleton, as obtained by our TBSS analysis because white matter regions were exclusively affected in del/+ animals. We used a Ranksum test a threshold of *p* < 0.05 to compare expression levels of the hemideleted genes in clusters stemming from the four contrasts of our TBSS analysis (increased FA in male del/+, decreased FA in male del/+, increased FA in female del/+, and decreased FA in female del/+) to expression levels over the entire FA skeleton.

For male animals, our analysis based on the Allen Mouse Brain Atlas found seven genes overexpressed in regions with increased FA and eight genes overexpressed with decreased FA in del/+. For female animals, we found three genes overexpressed in regions with increased FA and five genes overexpressed in regions with decreased FA in del/+. Three genes were only expressed in the male-specific endophenotype, i.e., in regions with FA increases in del/+ males: *Sez6l2*; *MVP*; and *TAOK2*.

For a complete overview over the identified genes, please refer to Table 2.

**Table 2:** Overview over genes identified as overexpressed in structurally altered brain regions of del/+ animals

Male del/+ > wild types	Male wild types > del/+	Female del/+ > wild types	Female wild types > del/+
*AI467606*	*4930451I11Rik*	*4930451I11Rik*	*4930451I11Rik*
*Gdpd3*	*AI467606*	*AI467606*	*AI467606*
*Kctd13*	*Asphd1*	*Kif22*	*Asphd1*
***MVP***	*Doc2a*		*Gdpd3*
*Qprt*	*Ino80e*		*Kctd13*
***Sez6l2***	*Ppp4c*		
***Taok2***	*Qprt*		
	*Spn*		

From left to right, columns provide the gene symbols for genes that were overexpressed in brain regions with (a) increased FA in male del/+, (b) decreased FA in male del/+, (c) increased FA in female del/+, and (d) decreased FA in female del/+. Highlighted in bold are the three genes that are only overexpressed in the male-specific endophenotype: *MVP*; *Sez6l2*; and *TAOK2*

## Discussion

Although several structural imaging studies have been published on human carriers^30,31,46,48,49^ and animal models^33–35^ of 16p11.2 hemideletion, sex-specific analyses of neuroanatomical changes have not yet been studied. Given that a variety of disorders associated with 16p11.2 hemideletion, such as ASD and ADHD, are strongly biased toward males, differential effects of a given CNV on the brain structure in male and female mutants could yield insight into the mechanisms that mediate increased male vulnerability. To the best of our knowledge, our findings are the first evidence for a sex-specific structural endophenotype in a mouse model of 16p11.2 hemideletion and they relate to our behavioral studies showing male-specific deficits in reward learning in 16p11.2 del mice^22^.

We used gene expression maps for the analysis of a structural animal imaging data set. This approach enabled us to develop an observer-independent hypothesis that genes associated with neurite outgrowth and the MAPK pathway might be relevant for sex-specific effects of 16p11.2 hemideletion on brain morphology. This notion is well in line with recent molecular findings from our lab that highlighted a sex-specific dysfunctional ERK signaling in male del/+ animals^22^.

### FA increases in medial fiber tracts constitute a male-specific structural endophenotype in a mouse model of 16p11.2 hemideletion

From published studies, a structural endophenotype in human 16p11.2 hemideletion carriers seems to emerge that is defined by white matter changes, namely FA increases, in the medial fiber tracts. Consistent findings have been reported in infantile carriers^46^, as well as in adults^30^ and in a mixed sample of adults and children^49^ with antagonistic effects of the hemideletion and hemiduplication^30,49^. These FA increases were interpreted in the context of other DTI parameters as potential evidence for reduced axonal fanning and crossing^46^. Reports of abnormally thick corpora callosum as one of the most frequent neuroradiological findings in deletion carriers also point to a relevance of 16p11.2 hemideletion for medial white matter structures^50^. These changes seem to be accompanied by gray matter increases in medial temporal, thalamic and cerebellar regions^30,31^, and most likely also in the basal ganglia^30^.

Structure–function relationships in the brain are certainly not straightforward and the fact that TBSS does not allow probabilistic inference on which gray matter regions fiber tracts originate from^41,42^ further complicates a functional interpretation of these findings. However, multiple lines of evidence support the idea of an involvement of peristriatal fiber tracts in these white matter changes as a neuroanatomical proxy of disrupted cortico-striatal circuits. Cortico-striatal networks are essential networks mediating reward sensitivity and outcome predictions^51,52^, functions that might be critically impaired in neurodevelopmental disorders such as ASD^53–55^ and ADHD^56–58^. A previous study of our lab has indeed demonstrated male-specific deficits in reward learning in the same mouse model that we also used for this imaging study^22^.

Of note, previous studies on mouse models of 16p11.2 hemideletion using MRI have reported structural^33,35^ or functional^34^ changes in the striatum. It should be noted that these studies have been criticized by some authors for pooling over genetically heterogeneous animals and comparatively large age ranges^33^. However, despite methodical differences between these studies, the convergence of findings within the striatum is highly remarkable and provides further evidence for a role for changes in this brain region in the pathophysiology of 16p11.2 hemideletion.

These peristriatal fiber tract changes might be driven by striatal volume increases during earlier changes of neurodevelopment. Intriguingly, mice modeling 16p11.2 hemideletion that were scanned at P7 were reported to show enlarged striatal regions^35^, whereas the animals scanned in our study at P70 did not show any significant structural changes within gray matter, although the *n* was low. It should be noted that third imaging study in mice of an unspecified age did not find any changes in the basal ganglia^34^. While methodical differences might well contribute to these discrepant findings, it seems most likely that mainly disturbed brain growth trajectories in mice modeling 16p11.2 hemideletion contribute to this heterogeneity. In humans, disturbed brain growth trajectories have been interpreted as a potentially central mechanism in neurodevelopmental disorders, since dynamic changes of brain structural anomalies, including the striatum, have been demonstrated in human subjects with ASD^28,59^.

Of note, also interhemispheric fibers contributed to the male-specific endophenotype, paralleling the findings in human hemideletion carriers^46,49,50^. Callosal FA changes also have been repeatedly implicated in various developmental disorders^60^ such as ASD^61,62^ and ADHD^62^. The direction of FA changes in these disorders, however, has been reported inconsistently, with most studies, e.g., in ASD patients pointing toward decreased, not increased FA values in callosal regions^62^. FA decreases in the corpus callosum were also reported to be associated with autistic traits across a sample of ASD and ADHD patients, as well as healthy controls, regardless of diagnosis^62^. In contrast, studies in toddlers with ASD repeatedly found FA increases in callosal regions^61,63,64^.

In contrast to this sex-specific endophenotype, del/+ animals of both sexes showed widespread FA decreases that appeared to be present in a variety of large fiber bundles such as the corpus callosum and external capsule. Given their widespread distribution over the brain, a potential interpretation as structural correlates of a specific behavioral deficit is difficult. While a wider range of behavioral domains seem to be impaired in male del/+, some behavioral deficits of the del/+, such as hyperactivity, are not sex-specific^23^. Accordingly, these behavioral changes in del/+ animals of both sexes might have structural correlates in some of these fiber tract changes that are shared between male and female mutants. However, since structural changes do not necessarily have to have functional consequences, this interpretation remains speculative.

### Genes overexpressed in regions of the male del/+ endophenotype are associated with neurite outgrowth and the MAPK pathway

We here have demonstrated that the ISH gene expression maps provided by the Allen Mouse Brain Atlas^37^ can be used to analyze structural animal imaging data sets and, thus, generate observer-independent hypotheses on transcriptomic changes in mutant mouse models. It stands to reason that genes are likely to be involved in the pathogenesis of brain structural changes if their expression patterns spatially overlap with these areas of structural change above chance, although our correlative approach certainly does not allow us to conclude that the detected gene expression patterns are actually the cause of the overlapping changes in neuroanatomy.

The Allen Mouse Brain Atlas provides a variety of gene expression maps that are, however, solely based on the brains of male, 56-day-old C57BL/6J mice^37^. Given the male-specific behavioral phenotypes and neuroanatomical endophenotype, this is a serious limitation, since differences in gene expression patterns between male and female animals can be reasonably expected. However, due to the limitations of the database, we were not able to conduct more detailed analyses with focus on sex-specific gene expression changes. To address this serious limitation, we will focus primarily on the genes that were exclusively overexpressed in the regions with increased FA in male mutants, i.e., the male-specific neuroanatomical endophenotype. While this focus certainly goes along with a potentially decreased sensitivity, it should help to reduce the likelihood of false positive findings.

Following this line of thought, we identified three genes overexpressed in regions with increased FA in male del/+: *TAOK2*; *Sez6l2*; and *MVP*. Although the number of these identified genes was too small to conduct a data-driven analysis of their biological functions, recent literature suggests a role for every of these three genes in the formation of neural circuits and—with varying degrees of evidence—an involvement in MAPK signaling. Their properties to influence the development and maintenance of neural networks renders the identified genes as plausible candidates to cause effects on white matter that are detectable by DW imaging. Of note, recent data from our lab have pointed to a male-specific regulation of ERK1 signaling in del/+ animals^22^.

Various evidence supports a role for TAOK2 in neurite outgrowth and spine maturation. In specific, TAOK2 has been linked to processes of basal dendrite formation and exon elongation in neurons. These processes require the phosphorylation of JNK^65^, a member of the MAPK pathway that does not activate ERK1/2^65–67^. Besides this involvement in neurite outgrowth, TAOK2 is also required for dendritic spine maturation and the stabilization of post-synaptic densities, namely PSD95. These effects are mediated via Septin 7^68^. Septin 7 is a member of a family of GTP-binding proteins that have the potential to interact with and to stabilize PSD95^68^, however, also have the ability to activate the MEK/ERK pathway at least in MDA-MB-231 human breast cancer cells^69^. Thus, Septin 7 activation might be a potential link of the TAOK2 pathway to ERK signaling.

Sez6l2 has also been implicated in the regulation of neurite outgrowth. Its expression is restricted to the CNS with highest expression rates in post-mitotic cortical layers, hippocampus, amygdala, and thalamus in the human fetal brain^70^. The Sez6l2 gene product exists both as membrane-standing protein and—after cleavage of its C-terminal transmembrane domain—as a secretable isoform sSez6l2. While the membrane-standing isoform suppresses neurite outgrowth, sSez6l2 induces this process^71^. Thus, a precise balance between the membrane-standing and the secretable isoform appears as important for a proper regulation of neurite outgrowth. Of note, Sez6l2 has been shown to phosphorylate protein kinase C (PKC)^72^, which is regarded as a likely downstream target to mediate these changes^71^. PKC, in turn, is an known activator of ERK1/2 via the MAPK pathway^73^. Consequently, also Sez6l2 signaling might depend upon the MAPK cascade, in general, and ERK activation, in specific.

While comparatively fewer studies on its role in neurite outgrowth have been conducted for MVP than the other two identified genes, its association with MAPK/ERK signaling is very well established. MVP has the potential to modulate ERK1 signaling^74–76^ and ERK1-induced transcriptional activity^77^, mostly by complexing with activated ERK and SHP2^74^. In mammalian brains, MVP is highly expressed in developing neurons and enriched especially in dendrites of cortical neurons. It binds to mRNAs, namely STEP^78^. STEP activation, in turn, regulates the phosphorylation of ERK^79^. These findings suggest two potential roles for MVP in neurons: regulation of synaptic plasticity and axonal transport mechanisms^79^. Disruption of the latter process might result in changes of axonal morphology that might be detectable by DW imaging.

As depicted, there are links to the MAPK pathway, in general, and ERK signaling, in specific, for all of the three genes, although the degrees of evidence vary admittedly. Importantly, ERK1 is one of the genes involved in the deletion, while this is not the case for ERK2^70,80,81^. The balance between phosphorylated ERK1 and ERK2 in the striatum profoundly influence reward-directed behavior^82,83^, with ERK1 signaling decreasing neuronal activity and the ability to associate reward and ERK2 exerting opposite effects^82,84^. Based on the observation that del/+ males showed sex-specific deficits in reward learning, a previous study of our lab determined levels of ERK1 and 2 phosphorylation in wild-type and del/+ animals at baseline and 40 min after sucrose intake that served as a natural reward^22^. At this time point, ERK striatal phosphorylation has been shown to be increased after reward delivery^85–87^. ERK1 phosphorylation was shown to be elevated in both wild-type and del/+ males, however, the elevation of ERK1 phosphorylation in del/+ males by far exceeded that in wild types. No evidence for similar pattern of ERK1 hyperphosphorylation was found in wild-type or del/+ females^22^. While these findings are certainly not a direct confirmation of our analyses based on gene expression maps, both studies highlight changes of ERK signaling as potentially critical for sex-specific functional and structural changes in del/+ animals. Findings of our recent study and our previous molecular work of our lab converge in another point. As illustrated above, STEP is a regulator of ERK phosphorylation^79^. Given the ERK1 hyperphosphorylation in del/+ males, the study of Grissom and colleagues followed up on potential changes of striatal STEP protein changes. Intriguingly, reduced levels of the STEP61 were found exclusively in del/+ males^22^, suggesting that elevated levels of phopshoERK1 were caused by a decreased ability to regulate dephosphorylation. MVP, which was implicated by our recent analyses, in turn, is a regulator of STEP expression and activity^78^. Our recent finding of a spatial overlap between brain structural changes and the expression patterns of the STEP62 regulator MVP fits well to the observed downregulation of STEP62 in del/+ males. Our data cannot provide a mechanistic explanation, however, it further corroborates the notion of a dysregulated ERK phosphorylation as a causal factor for both the sex-specific structural endophenotype as well as behavioral anomalies.

### Limitations

There are several limitations in this study. First, animals that underwent MR imaging were not the mice that were used for the behavioral testing. While it would certainly be preferable to use the same animals for both experiments, the pronounced effects on both behavior and brain structure in our model of 16p11.2 hemideletion strongly suggest that both changes are robust.

Second, we have only scanned a comparatively small number of animals per group. Although the effects observed in our analysis are correctable by TFCE, a higher *n* would be preferable. Using larger amounts of data sets will be an important goal for future studies. However, given the standardized environmental conditions during their upbringing and the homogeneous genetic background of the inbred strains used, it should be possible to robustly detect the effects of chromosomal variation on brain structure. It should also be noted that robust behavioral effects in del/+ animals were observed using comparable group sizes.

The mice used in this study are on a mixed 129/B6 genetic background due to partial lethality of the 16p11.2 deletion on a pure B6 background. Importantly, we used littermate controls for our MRI study. However, variations in the genetic background between the mutant mice and the littermate controls has been suggested to potentially contribute to the observed phenotype^88^, as not only the mutation but also flanking segments stemming from the genetic background of the embryonic stem (ES) cells used to make the mutant will be transmitted to a model organism. These effects are most pronounced in models with homozygous mutations^89–91^. In our hemideleted model, flanking segments from the ES cells used to make the mutation will be present in only one locus, making its impact much less pronounced.

We have here used gene expression atlases as a novel tool to develop observer-independent hypotheses. While the Allen Mouse Brain Atlas provides a high-resolution database of gene expression in the C57BL/6J mice, it certainly provides only gene expression data of one mouse strain at P56^37^. Consequently, gene expression might vary across strains and ages. Importantly, in this context, the gene expression maps of the Allen Mouse Brain Atlas were determined in male mice only. Gene expression in the mouse brain has been shown to vary due to sex^92^. Thus, the lack of gene expression maps derived from the brains of female animals appears certainly as a critical limitation of this approach, especially with regard to sex-specific analyses. Although this is an important constraint, it deserves to be pointed out that we focused on a male-specific endophenotype. This should help to confine potential distortions of our results due to sex-specific differences in gene expression. It should also be pointed out that the findings of our gene expression atlas-based analysis converged with the results of an earlier molecular study of our lab on the same mouse model.

Although changes in gene expression might well influence brain structure long after the embryonic period, it should be noted that a comparison of brain structural changes with gene expression maps acquired at P56 might not necessarily allow inference on the pathogenesis of brain structural changes. The relationship between neuroanatomical changes and spatially overlapping gene expression patterns could also reflect the consequence rather than the causes of these neuroanatomical changes. These changes in gene expression could, for example, lead to changes in the MAPK pathway that are functionally reflected by male impairments in reward learning^22^.

These limitations emphasize that analyses based on gene expression atlases are solely suitable for the generation of hypotheses that should be tested in subsequent molecular studies. Thus, characterizing changes of MAPK signaling in structurally altered regions will be an important aim for future studies.

Human data on increased male vulnerability still are relatively scarce. A previous study has shown a preponderance of 1.3 male to 1 female carrier of 16p11.2 hemideletion in ASD and 1.6 male to 1 female carriers in ID^20^, but it should be noted that the male excess was comparatively small for both disorders. Further studies will be needed to corroborate the notion that 16p11.2 hemideletion is more common in males. Despite these caveats, we regard these findings as encouraging hints at a translational relevance for our own findings of male-specific effects of 16p11 hemideletion in mouse models.

## Conclusion and outlook

Here we have demonstrated that a mouse model of 16p11.2 hemideletion with male-specific deficits in reward learning also exhibits a sex-specific endophenotype with FA increases in peristriatal and medial fiber tracts in male del/+ only. Analyses based on the Allen Mouse Brain Atlas showed that genes overexpressed in structurally altered regions were associated with neurite outgrowth and the MAPK pathway, with the latter already shown to be dysregulated in this mouse model. A better characterization of dysfunctional MAPK signaling in mice modeling 16p11.2 hemideletion will be an important aim for future studies.
